# Diuretic Enhanced Ultrasonography in the Diagnosis of Pyeloureteral Obstruction

**DOI:** 10.3390/medicina55100670

**Published:** 2019-10-03

**Authors:** Vytis Kazlauskas, Andrius Cekuolis, Vytautas Bilius, Marius Anglickis, Gilvydas Verkauskas

**Affiliations:** 1 Clinic of Gastroenterology, Nephrourology and Surgery, Institute of Clinical Medicine, Faculty of Medicine, Vilnius University, 08406 Vilnius, Lithuania; vytautas.bilius@santa.lt (V.B.); gilvydas.verkauskas@santa.lt (G.V.); 2 Clinic of Radiology, Vilnius University, 08406 Vilnius, Lithuania; andrius.cekuolis@santa.lt; 3 Clinic of Surgery and Vascular Surgery, Department of Urology, Vilnius City Clinical Hospital, 10007 Vilnius, Lithuania; mmariusa@gmail.com

**Keywords:** diuretic ultrasonography, hydronephrosis, pyeloureteral junction obstruction, pyeloplasty, diuretic renal scan

## Abstract

*Background and Objectives*: To determine the value of diuretic ultrasonography for the diagnosis of obstructive hydronephrosis. *Materials and Methods*: Diuretic enhanced ultrasonography was used routinely as a part of examination of patients with hydronephrosis in our Department. There were 72 patients (42 males, 30 females; aged 2 months to 17 years; median age 7.07 years) with a sonoscopic diagnosis of hydronephrosis included from January 2006 until October 2011. The anteroposterior diameter (AD) of renal pelvis was measured sonoscopically before and at sixty minutes after furosemide injection. A weight-adjusted dose of 1 mg/kg of furosemide was administered intravenously. *Results*: Patients were operated on if pyeloureteral obstruction was suspected because of low or deteriorating differential renal function, increasing hydronephrosis or symptoms thereof. Hydronephrosis was unilateral in 61 (84.7%) and bilateral in 11 (15.3%) patients. The median AD of pelvis before furosemide injection was 22 mm in operated and 17 mm in non-operated patients (*p* = 0.005). Sixty minutes after furosemide injection, the AD of pelvis in operated patients was 35.5 mm and 25.8 mm in non-operated—25.8 mm (*p* < 0.001). Logistic regression model demonstrated that significant factors for surgery were: AD 60 min after furosemide infection and ultrasonographic parenchymal sclerosis. *Conclusion*: Ultrasound measurement of the AD of renal pelvis 1 h after the injection of furosemide used as an additional investigation can help in predicting obstructive hydronephrosis.

## 1. Introduction

The most common abnormal finding in routine antenatal ultrasonography is a dilatation of the renal collecting system [1]. This finding is of uncertain clinical significance, ranging from transient dilatation to deleterious obstructive uropathies. The majority of prenatally detected hydronephroses demonstrate spontaneous resolution or remain clinically silent, and the rest (about 25%) show progression and require surgical management [2,3]. The most frequent reason of progressing hydronephrosis is a narrowing or kinking of the ureter at the pyeloureteral junction, causing obstruction to urinary flow [4]. Pyeloureteral junction obstruction (PUJO) may cause pain, pyelonephritis, stones and deterioration of renal function [5]. Nowadays, radiological investigation is still irreplaceable to differentiate obstructed from non-obstructed hydronephrosis and to justify surgical intervention. Society for Fetal Urology (SFU) provides a hydronephrosis grading system where grades 0–4 reflect ultrasound features of renal collecting system and parenchyma assuming pelvi-calyceal dilatation and thinning of parenchyma [6]. However, collecting system dilatation may be non-obstructive and the parenchymal thickness does not always reflect renal function. Therefore, diuretic renal scan (DRS) is the most applicable diagnostic option for PUJO, followed by modern diagnostic equipment such as magnetic resonance urography or computed tomography with an additional value in visualizing adjacent anatomy [5]. Diagnosis of obstruction by means of DRS is still considered the golden standard of investigation [7]. However, DRS is associated with radiation exposure and high cost. In addition, most toddlers cannot lie still for a proper DRS and require sedation. One of the alternative tools to detect congenital ureteral obstruction without radiation is an increase of the anteroposterior diameter (AD) on serial ultrasound scans. However, recently it has been criticized as having a weak correlation to the deterioration of split renal function (SRF) in high grade hydronephrosis [8]. Diuretic enhanced ultrasound pelvi-caliceal measurements are used routinely in some centers, but to our knowledge have not been studied systematically [5]. The purpose of this study was to review our experience with diuretic enhanced ultrasonoscopy and to determine whether it is superior to conventional ultrasound when opting for pyeloplasty.

## 2. Materials and Methods

**Figure 1:**
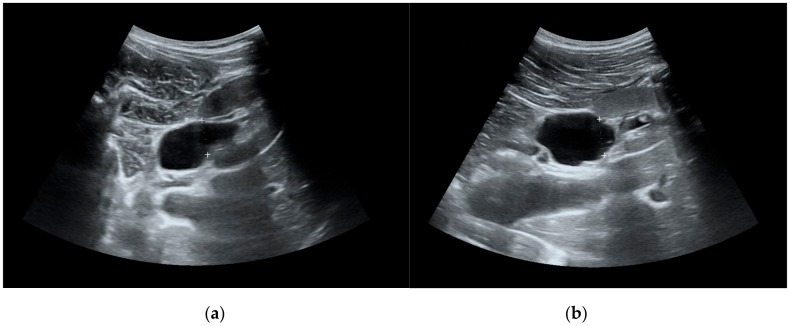
(**a**) Renal pelvis before furosemide injection. (**b**) Renal pelvis 60 min after furosemide injection.

Seventy-two patients (42 males, 30 females; aged 2 months to 17 years; median age 7.07 years) with ultrasonographic diagnosis of hydronephrosis (AD cut-off value 10 mm) were included from January 2006 until October 2011 and followed for the median of 7.5 (1.5, 24) months. The linear regression model demonstrated that patient age was a confounding factor for the AD, therefore we divided patients into 3 separate groups (0–4 years, 5–9 years and 10–17 years). All children underwent a detailed clinical examination and measurement of serum urea and creatinine, and a radiological investigation including voiding cystourethrogram. Patients with reflux or neurogenic urinary bladder were excluded. A weight-adjusted dose of 1 mg/kg of furosemide was administered intravenously (maximum dose of 20 mg) after adequate oral hydration. Diuretic enhanced ultrasonography was performed in all patients only once in the beginning of follow-up. The AD of renal pelvis was measured and recorded before and at 60 min after furosemide injection. (Figure 1) 

Ultrasonografic diagnosis of parenchymal sclerosis was considered when thinning of parenchyma and/or parenchymal hyperechogenicity was detected. Indications for pyeloplasty was progressive dilatation on follow-up ultrasound scans, worsening of differential renal function on DRS or symptoms thereof (lumbar pain or pyelonephritis). Statistical analysis of ultrasonographic risk factors for pyeloplasty was performed using R commander version 2.5–1 (McMaster University, Hamilton, Ontario, Canada (GNU General Public License)). Covariates in the logistic regression model for operation where: AD before diuretic, AD after diuretic and parenchymal status. The level of significance was assumed when α < 0.05. Ultrasonoscopy was done by one investigator who was independent of the decision for pyeloplasty. We used a Medison Accuvix XQ ultrasound device (Medison Americal, Cypress, California, US) for measurements. Institutional review board permission was obtained for the analysis of retrospective data. The study was approved by the Vilnius regional bioethics committee (158200-18/6-1044-544, the date of approval 05/06/2018).

## 3. Results

Hydronephrosis was unilateral in 61 (84.7%) and bilateral in 11 (15.3%) patients. The total number of renal units (RU) with hydronephrosis was 83. The total number of evaluations of RU acceptable for the study was 144. In unilateral cases, hydronephrosis was left-sided in 49.2% (*n* = 30) and right-sided in 50.8% (*n* = 31). 28 (39%) of patients who were operated on (all of them on one side) and the remaining 44 (61%) patients were followed-up. The overall median AD before diuretic was 22 (17, 21.8) in operated and 17 (12.9, 20.5) in non-operated patients (*p* = 0.005) and after diuretic 35.5 (29.2, 39.2) in operated patients and 25.8 (19.8, 29) in non-operated patients (*p* < 0.001). Patient and AD descriptive statistics according to age are shown in Table 1.

There were contralateral, primarily non-dilated RU which responded to furosemide injection by dilatation and dilated ones which did not change (Table 2). 

Spearman’s rank order correlation test (Spearman’s rank correlation coefficient defined as *rho*) demonstrated a negative correlation between primary AD and its increase after furosemide injection *rho* = −0.47 (*p* < 0.001) and a positive correlation between increase in AD of a dilated kidney and contralateral dilatation of a healthy kidney when present (*rho* = 0.51, *p* = 0.04) 

AD of primarily dilated kidneys before and after furosemide injection were not influenced by the presence of contralateral dilatation *p* = 0.63.

**Figure 2:**
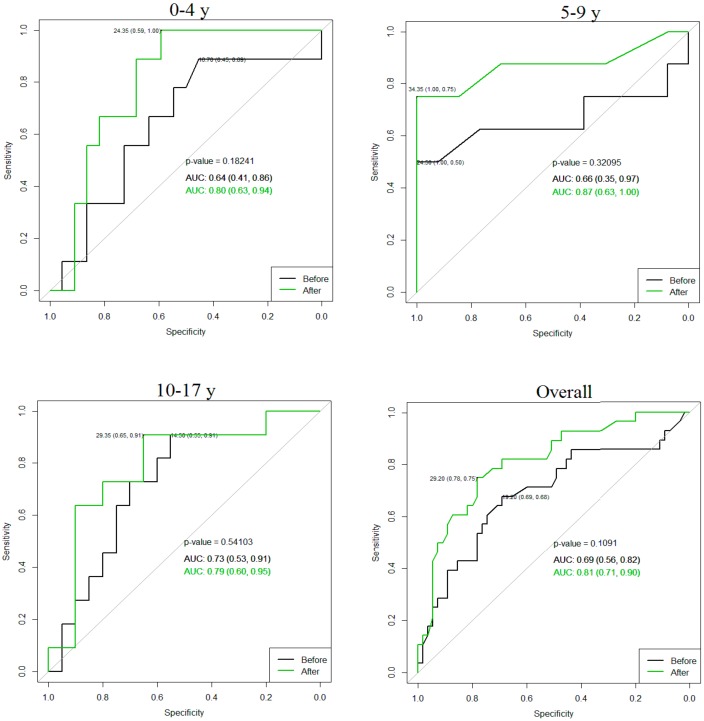
Value of anteroposterior diameter (AD) for the risk of surgery. Green receiver operating characteristic (ROC) curve after furosemide adjusted by age. Y: Years, AUC: Area under the curve.

The logistic regression model and a stepwise model selection demonstrated significant predictors for operation in age groups: In the 0–4 year group—AD after furosemide injection (*p* = 0.04), in the 5–9 year group—parenchymal sclerosis (*p* = 0.02) and the AD after furosemide was close to the level of significance (*p* = 0.05) and in the 10–17 year group—parenchymal sclerosis (*p* = 0.02). AD after diuretic cut-off value for operation: In the 0–4 year group 24.35 mm (sensitivity 1, specificity 0.59); in the 5–9 year group 34.35 mm (sensitivity 0.75, specificity 1), in the 10–17 year group 29.35 mm (sensitivity 0.91, specificity 0.65) and overall 29.2 mm (sensitivity 0.78, specificity 0.75). Receiver operating characteristic (ROC) curve comparisons with and without diuretic are demonstrated in Figure 2.

## 4. Discussion

It is important to define the group of investigated children as a not “evidently” surgical group. Collecting cases retrospectively, the selection bias is unavoidable: In our study it is constituted of children with incidental ultrasonographic findings, those already followed because of symptoms or prenatal cases. Natural history of hydronephrosis is better understood following prospective studies [9,10,11,12]. Despite this, there is no golden standard of investigation to decide which kidney is at risk of deterioration if no surgery is undertaken. Apparently, even infants with isolated unilateral high-grade hydronephrosis (SFU 3–4) might be managed conservatively [5,9]. Indications for operation at follow-up are worsening of hydronephrosis, deterioration of SRF by more than 10%, SRF below 40% with an obstructive curve in DRS or recurrent febrile urinary tract infection [13]. Neither poor drainage, nor decreased renal function on DRS can be considered as absolute indications for operation. Moreover, no controlled studies have shown that the kidney with poor function is more at risk of deterioration than the kidney with normal SRF [14,15]. Yet several studies expressed concern about ongoing, irreversible loss of renal function following delayed pyeloplasty and support early surgical management [16,17]. Functional improvement, in the presence of low SRF at entry, has been observed after surgery only in a part of patients. Kumar M et al. reports >5% improvement in low SRF (<40%) after pyeloplasty in 41–46% of patients retaining improved renal function after 5 years of follow-up [18]. Another study demonstrated significant increase in SRF in 75% of children whose SRF was impaired preoperatively, but just in early renograms (7–9 weeks postoperatively) [19]. Surgical indications still remain extremely different, varying from active surveillance and delayed pyeloplasty to early surgical management [20,21]. An absence of well-controlled prospective studies in this field is partly responsible for this situation [22,23]. The searches for early predictors of renal function deterioration have shown that increased cortical transit time on MAG3 renal scans (more than 3 min) can be a promising early predictor of ongoing obstruction, renal function deterioration and surgery [24]. However, this method supposes radiation exposure, often requires sedation for children and is more expensive and less available in small centers and ambulatory settings. Various ultrasound modalities have been suggested to replace DRS in prediction of renal deterioration and surgery. Fetal AD, SFU grade (3–4) and AD parenchyma thickness ratio can be considered as the earliest instruments to indicate surgery [25,26]. Postnatal ultrasound cut-off values for renal obstruction on DRS have been suggested to be 15–20 mm AD [27]. Onen’s alternative grading system, of grades 3 or 4 with no jets on a Doppler scan, increased sensitivity to 78.9% and accuracy to 92% in diagnosing obstruction [28]. Prospective study has shown that the parenchyma/hydronephrosis area ratio has been more accurate in predicting obstruction in comparison to SFU grade and AD, but requires specific software for calculations [29]. Diuretic ultrasonography is fast, cheap and simple to test, which could add to the armamentarium of urologist as an additional tool, when taking into account history and clinical picture. Correlation between change in renal length after intravenous diuretic administration and functional status was demonstrated previously [30]. We can also hypothesize, from our data, that reaction to furosemide can be investigated as a potential surrogate marker for differential renal function. Limitation of our study is its retrospective nature and small sample size. Also, indications for operation were not always justified by DRS. Therefore, we were unable to investigate correlations between diuretic response and functional renal status. Indications for pyeloplasty in our study were progressive dilatation and worsening of renal function or symptoms thereof (lumbar pain or pyelonephritis). Measurements, 30 min after diuretic administration, were also performed in some of our children, but not consistently and, in our opinion, persistent dilatation after 1 h is more informative. A longer interval between diuretic administration and ultrasound scan enables better rejection of false positive cases, as we noticed that even healthy kidneys may demonstrate delayed reaction to diuretic. Rundstedt et al. discuss that tracer clearance on a MAG3 scan at 40 min after injection is a more sensitive indicator of obstruction than that of a standard T1/2 [31]. According to this article, we consider that a reasonable time to wait until the urine overload leaks out of a healthy collecting system after diuretic stimulation may be even more than 1 h (taking into account the peak of furosemide effect after 30 min of injection) [32]. This can explain the fact that even some healthy kidneys demonstrate dilatation after 1 h.

The logistic regression model demonstrated that significant risk factors for surgery were: AD 1 h after furosemide injection and parenchymal sclerosis. Hydronephrosis side and contralateral response to furosemide were not significant risk factors for surgery, however their *p*-values were close to the level of significance and may be discussed in future studies. We also detected that AD before furosemide injection had negative correlation to AD increase after furosemide injection. This result is probably caused by the selection-high prevalence of older children. Initially non-dilated kidneys demonstrated greater increase in AD when compared to hydronephrotic side. We have also observed that non-sclerotized kidneys demonstrated greater increase after furosemide than sclerotized, however there was no significant difference. In the 10–17 year group, both AD before and after furosemide demonstrated statistically significant difference in both operated and non-operated patients, revealing no evidence of additional diagnostic value of diuretic enhanced ultrasonoscopy in this group.

The aim of this study was to compare the efficacy of conventional ultrasound with diuretic enhanced modality in the diagnosis and follow-up of hydronephrosis, but not to compare its efficacy to DRS. Diuretic enhanced ultrasonoscopy, combining AD and parenchymal evaluation, might be a good alternative for the selection of surgical cases, especially in an ambulatory setting, where no device for DRS is available, or when parents refuse radiation exposure or general anesthesia in small children

## 5. Conclusions

Furosemide testing, used as an additional investigation, may help in the work-up of patients with hydronephrosis. AD after diuretic in children up to four years old, one hour after furosemide injection, was a significant risk factor for operation.

## Figures and Tables

**Table 1:** Medians (quartiles in brackets) of anteroposterior diameter (AD) before and after diuretic with respect to age and operative management.

Age (Years) *N* = 83	Diuretic Status	Op (Pelvis AD mm)	Non-Op (Pelvis AD mm)	*p*-Value
0–4	Before	22.0 (18.20, 25.900)	17.5 (13.85, 22.625)	0.1115
Op 9 (29%)	After	29.3 (26.0, 33.000)	23.5 (18.3, 27.675)	0.003886 *
Non-op 22 (71%)	Increase of AD 60 min after	8.0 (6.200, 9.60)	4.2 (0.025, 6.85)	0.07684
5–9	Before	23.0 (14.3, 35.25)	17.8 (13.5, 18.20)	0.1157
Op 8 (38%)	After	39.5 (35.75, 43.7)	26.0 (20.00, 29.0)	0.00285 *
Non-op 13 (62%)	Increase of AD 60 min after	4 (2.75, 26.4)	8 (4.00, 11.0)	0.572
10–17	Before	23.40 (19.525, 28.100)	13.75 (10.875, 21.425)	0.01313 *
Op 11 (35%)	After	37.15 (32.8, 39.20)	27.55 (20.4, 32.25)	0.003213 *
Non op 20 (55%)	Increase of AD 60 min after	12.60 (7.0; 16.300)	9.35 (6.9; 12.325)	0.1603

* *p* < 0.05 was assumed as statistically significant.

**Table 2:** Reaction to furosemide injection with respect to primary AD dilatation.

*N* = 144	Primarily Dilated RU	Primarily Non-Dilated RU	Chi-Squared Test	*p*-Value
Response to furosemide	71 (86%)	16 (22%)	51.722	<0.001
No response to furosemide	12 (14%)	45 (74%)		
Odds of reaction to furosemide	5.92	0.36		

RU: Renal units.
